# Author Correction: Twelve-month specific IgG response to SARS-CoV-2 receptor-binding domain among COVID-19 convalescent plasma donors in Wuhan

**DOI:** 10.1038/s41467-021-25109-1

**Published:** 2021-08-05

**Authors:** Cesheng Li, Ding Yu, Xiao Wu, Hong Liang, Zhijun Zhou, Yong Xie, Taojing Li, Junzheng Wu, Fengping Lu, Lu Feng, Min Mao, Lianzhen Lin, Huanhuan Guo, Shenglan Yue, Feifei Wang, Yan Peng, Yong Hu, Zejun Wang, Jianhong Yu, Yong Zhang, Jia Lu, Haoran Ning, Huichuan Yang, Daoxing Fu, Yanlin He, Dongbo Zhou, Tao Du, Kai Duan, Demei Dong, Kun Deng, Xia Zou, Ya Zhang, Rong Zhou, Yang Gao, Xinxin Zhang, Xiaoming Yang

**Affiliations:** 1 Sinopharm Wuhan Plasma-derived Biotherapies Co., Ltd, Wuhan, China; 2 Chengdu Rongsheng Pharmaceuticals Co., Ltd, Chengdu, China; 3 Beijing Tiantan Biological Products Co., Ltd, Beijing, China; 4 Wuhan Institute of Biological Products Co. Ltd, Wuhan, China; 5 China National Biotec Group Company Limited, Beijing, China; 6 Research Laboratory of Clinical Virology, Ruijin Hospital and Ruijin Hospital North, National Research Center for Translational Medicine, Shanghai Jiao Tong University of Medicine, Shanghai, China

**Keywords:** Adaptive immunity, Antibodies, Antimicrobial responses, Viral infection

Correction to: *Nature Communications* 10.1038/s41467-021-24230-5, published online 6 July 2021.

The original version of this Article contained an error in the abstract, which incorrectly read ‘The level of RBD-IgG decreases with time, with the titer stabilizing at 64.3% of the initial level by 9 month’. The correct version replaces this sentence with ‘The level of RBD-IgG decreases with time, with the titer stabilizing at 35.7% of the initial level by the 9th month’. This has been corrected in both the PDF and HTML versions of the Article.

